# Equivalence Tests: A Practical Primer for *t* Tests, Correlations, and Meta-Analyses

**DOI:** 10.1177/1948550617697177

**Published:** 2017-05-05

**Authors:** Daniël Lakens

**Affiliations:** 1 Human Technology Interaction Group, Eindhoven University of Technology, Eindhoven, the Netherlands

**Keywords:** research methods, equivalence testing, null hypothesis significance testing, power analysis

## Abstract

Scientists should be able to provide support for the absence of a meaningful effect. Currently, researchers often incorrectly conclude an effect is absent based a nonsignificant result. A widely recommended approach within a frequentist framework is to test for *equivalence*. In equivalence tests, such as the two one-sided tests (TOST) procedure discussed in this article, an upper and lower equivalence bound is specified based on the smallest effect size of interest. The TOST procedure can be used to statistically reject the presence of effects large enough to be considered worthwhile. This practical primer with accompanying spreadsheet and R package enables psychologists to easily perform equivalence tests (and power analyses) by setting equivalence bounds based on standardized effect sizes and provides recommendations to prespecify equivalence bounds. Extending your statistical tool kit with equivalence tests is an easy way to improve your statistical and theoretical inferences.

Scientists should be able to provide support for the null hypothesis. A limitation of the widespread use of traditional significance tests, where the null hypothesis is that the true effect size is zero, is that the absence of an effect can be rejected, but not statistically supported. When you perform a statistical test, and the outcome is a *p* value larger than the α level (e.g., *p* > .05), the only formally correct conclusion is that the data are not surprising, assuming the null hypothesis is true. It is not possible to conclude there is no effect when *p* > α—our test might simply have lacked the statistical power to detect a true effect.

It is statistically impossible to support the hypothesis that a true effect size is exactly zero. What *is* possible in a frequentist hypothesis testing framework is to statistically reject effects large enough to be deemed worthwhile. When researchers want to argue for the absence of an effect that is large enough to be worthwhile to examine, they can test for *equivalence* (Wellek, 2010). By rejecting an effect (indicated in this article by Δ) more extreme than predetermined lower and upper equivalence bounds (−Δ*_L_* and Δ*_U_*, e.g., effect sizes of Cohen’s *d* = −.3 and *d* = .3), we can act as if the true effect is close enough to zero for our practical purposes. Equivalence testing originates from the field of pharmacokinetics (Hauck & Anderson, 1984), where researchers sometimes want to show that a new cheaper drug works just as well as an existing drug (for an overview, see Senn, 2007, Chapters 15 and 22). A very simple equivalence testing approach is the “two one-sided tests” (TOST) procedure (Schuirmann, 1987). In the TOST procedure, an upper (Δ*_U_*) and lower (−Δ*_L_*) equivalence bound is specified based on the smallest effect size of interest (SESOI; e.g., a positive or negative difference of *d* = .3). Two composite null hypotheses are tested: H0_1_: Δ ≤ −Δ*_L_* and H0_2_: Δ ≥ Δ*_U_*. When both these one-sided tests can be statistically rejected, we can conclude that −Δ*_L_* < Δ < Δ*_U_* or that the observed effect falls within the equivalence bounds and is close enough to zero to be practically equivalent (Seaman & Serlin, 1998).

Psychologists often incorrectly conclude there is no effect based on a nonsignificant test result. For example, the words “no effect” had been used in 108 articles published in *Social Psychological and Personality Science* up to August 2016. Manual inspection revealed that in almost all of these articles, the conclusion of “no effect” was based on statistical nonsignificance. Finch, Cumming, and Thomason (2001) reported that in the *Journal of Applied Psychology*, a stable average of around 38% of articles with nonsignificant results accept the null hypothesis. This practice is problematic. With small sample sizes, nonsignificant test results are hardly indicative of the absence of a true effect, and with huge sample sizes, effects can be statistically significant but practically and theoretically irrelevant. Equivalence tests, which are conceptually straightforward, easy to perform, and highly similar to widely used hypothesis significance tests that aim to reject a null effect, are a simple but underused approach to reject the possibility that an effect more extreme than the SESOI exists (Anderson & Maxwell, 2016).

Psychologists would gain a lot by embracing equivalence tests. First, researchers often incorrectly use nonsignificance to claim the absence of an effect (e.g., “there were no gender effects, *p* > .10”). This incorrect interpretation of *p* values would be more easily recognized and should become less common in the scientific literature if equivalence tests were better known and more widely used. Second, where traditional significance test only allows researchers to reject the null hypothesis, science needs statistical approaches that allow us to conclude meaningful effects are absent (Dienes, 2016). Finally, the strong reliance on hypothesis significance tests that merely aim to reject a null effect does not require researchers to think about the effect size under the alternative hypothesis. Exclusively focusing on rejecting a null effect has been argued to lead to imprecise hypotheses (Gigerenzer, 1998). Equivalence testing invites researchers to make more specific predictions about the effect size they find worthwhile to examine. Bayesian methods can also be used to test a null effect (e.g., Dienes, 2014), but equivalence tests do not require researchers to switch between statistical philosophies to test the absence of a meaningful effect, and the availability of power analyses for equivalence tests allows researchers to easily design informative experiments.

There have been previous attempts to introduce equivalence testing to psychology (Quertemont, 2011; Rogers, Howard, & Vessey, 1993; Seaman & Serlin, 1998). I believe there are four reasons why previous attempts have largely failed. First, there is a lack of easily accessible software to perform equivalence tests. To solve this problem, I’ve created an easy to use spreadsheet and R package to perform equivalence tests for independent and dependent *t* tests, correlations, and meta-analyses (see https://osf.io/q253c/) based on summary statistics. Second, in pharmacokinetics, the equivalence bounds are often defined in raw scores, whereas it might be more intuitive for researchers in psychology to express equivalence bounds in standardized effect sizes. This makes it easier to perform power analyses for equivalence tests (which can also be done with the accompanying spreadsheet and R package) and to compare equivalence bounds across studies in which different measures are used. Third, there is no single article that discusses both power analyses and statistical tests for one-sample, dependent and independent *t* tests, correlations, and meta-analyses, which are all common in psychology. Finally, guidance on how to set equivalence boundaries has been absent for psychologists, given that there are often no specific theoretical limitations on how small effects are predicted to be (Morey & Lakens, 2017) nor cost–benefit boundaries of when effects are too small to be practically meaningful. This is a chicken–egg problem, since using equivalence tests will likely stimulate researchers to specify which effect sizes are predicted by a theory (Weber & Popova, 2012). To bootstrap the specification of equivalence bounds in psychology, I propose that when theoretical or practical boundaries on meaningful effect sizes are absent, researchers set the bounds to the smallest effect size they have sufficient power to detect, which is determined by the resources they have available to study an effect.

## Testing for Equivalence

In this article, I will focus on the TOST procedure (Schuirmann, 1987) of testing for equivalence because of its simplicity and widespread use in other scientific disciplines. The goal in the TOST approach is to specify a lower and upper bound, such that results falling within this range are deemed equivalent to the absence of an effect that is worthwhile to examine (e.g., Δ*_L_* = −.3 to Δ*_U_* = .3, where Δ is a difference that can be defined by either standardized differences such as Cohen’s *d* or raw differences such as .3 scale point on a 5-point scale). In the TOST procedure, the null hypothesis is the *presence* of a true effect of Δ*_L_* or Δ*_U_*, and the alternative hypothesis is an effect that falls within the equivalence bounds or the *absence* of an effect that is worthwhile to examine. The observed data are compared against Δ*_L_* and Δ*_U_* in two one-sided tests. If the *p* value for both tests indicates the observed data are surprising, assuming Δ*_L_* or Δ*_U_* are true, we can follow a Neyman–Pearson approach to statistical inferences and reject effect sizes larger than the equivalence bounds. When making such a statement, we will not be wrong more often, in the long run, than our Type 1 error rate (e.g., 5%). It is also possible to test for inferiority, or the hypothesis that the effect is smaller than an upper equivalence bound, by setting the lower equivalence bound to ∞.^1^ Furthermore, equivalence bounds can be symmetric around zero (Δ*_L_* = −.3 to Δ*_U_* = .3) or asymmetric (Δ*_L_* = −.2 to Δ*_U_* = .4).

**Figure 1.**
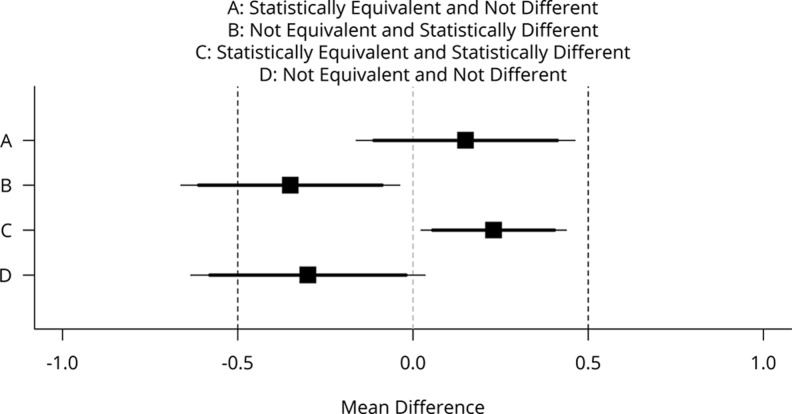
Mean differences (black squares) and 90% confidence intervals (CIs; thick horizontal lines) and 95% CIs (thin horizontal lines) with equivalence bounds Δ*_L_* = −.5 and Δ*_U_* = .5 for four combinations of test results that are statistically equivalent or not and statistically different from zero or not.

When both null hypothesis significance tests (NHST) and equivalence tests are used, there are four possible outcomes of a study: The effect can be statistically equivalent (larger than Δ*_L_*, smaller than Δ*_U_*) and not statistically different from zero, statistically different from zero but not statistically equivalent, statistically different from zero and statistically equivalent, or undetermined (neither statistically different from zero nor statistically equivalent). In Figure 1, mean differences (black squares) and their 90% (thick lines) and 95% confidence intervals (CIs; thin lines) are illustrated for four scenarios. To conclude equivalence (Scenario A), the 90% CI around the observed mean difference should exclude the Δ*_L_* and Δ*_U_* values of −.5 and .5 (indicated by black vertical dashed lines).^2^

The traditional two-sided null hypothesis significance test is rejected (Scenario B) when the CI around the mean difference does not include 0 (the vertical gray dotted line). Effects can be statistically different from zero *and* statistically equivalent (Scenario C) when the 90% CI exclude the equivalence bounds and the 95% CI exclude zero. Finally, an effect can be undetermined, or not statistically different from zero, and not statistically equivalent (Scenario D) when the 90% CI includes one of the equivalence bounds and the 95% CI includes zero.

In this article, the focus lies on the TOST procedure, where two *p* values are calculated. Readers are free to replace decisions based on *p* values by decisions based on 90% CIs if they wish. Formally, hypothesis testing and estimation are distinct approaches (Cumming & Finch, 2001). For example, while sample size planning based on CIs focusses on the width of CIs, sample size planning for hypothesis testing uses power analysis to estimate the probability of observing a significant result (Maxwell, Kelley, & Rausch, 2008). Since the TOST procedure is based on a Neyman–Pearson hypothesis testing approach to statistics, and I’ll explain how to calculate the tests as well as how to perform power analysis, I’ll focus on the calculation of *p* values for conceptual consistency.

## Equivalence Tests for Differences Between Two Independent Means

The TOST procedure entails performing two one-sided tests to examine whether the observed data are surprisingly larger than an equivalence boundary lower than zero (Δ*_L_*) or surprisingly smaller than an equivalence boundary larger than zero (Δ*_U_*). The equivalence test assuming equal variances is based on:

1 $$t_{\text{L}}=\frac{{\bar{M}}_{1}-{\bar{M}}_{2}-\Delta_{\text{L}}}{\text{σ}\sqrt{\frac{1}{n_{1}}+\frac{1}{n_{2}}}}\text{ and }t_{\text{U}}=\frac{{\bar{M}}_{1}-{\bar{M}}_{2}-\Delta_{\text{U}}}{\text{σ}\sqrt{\frac{1}{n_{1}}+\frac{1}{n_{2}}}},$$

where *M*_1_ and *M*_2_ indicate the means of each sample, *n*_1_ and *n*_2_ are the sample size in each group, and σ is the pooled standard deviation (*SD*):

2 $$\sigma =\sqrt{\frac{\left(n_{1}-1\right)SD_{\text{1}}^{2}+\left(n_{2}-1\right)SD_{\text{2}}^{2}}{n_{1}+\;n_{2}-2}}.$$

Even though Student’s *t* test is by far the most popular *t* test in psychology, there is general agreement that whenever the number of observations are unequal across both conditions, Welch’s *t* test (1947), which does not rely on the assumption of equal variances, should be performed by default (Delacre, Lakens, & Leys, 2017; Ruxton, 2006). The equivalence test not assuming equal variances is based on:

3 $$t_{\text{L}}=\frac{{\bar{M}}_{1}-{\bar{M}}_{2}-\Delta_{\text{L}}}{\sqrt{\frac{SD_{\text{1}}^{2}}{n_{1}}+\frac{SD_{\text{2}}^{2}}{n_{2}}}}\text{ and }t_{\text{U}}=\frac{{\bar{M}}_{1}-{\bar{M}}_{2}-\Delta_{\text{U}}}{\sqrt{\frac{SD_{\text{1}}^{2}}{n_{1}}+\frac{SD_{\text{2}}^{2}}{n_{2}}}},$$

where the degrees of freedom (*df*) for Welch’s *t* test are based on the Sattherthwaite (1946) correction:

4 $$df_{\text{w}}=\frac{{\left(\frac{SD_{\text{1}}^{2}}{n_{1}}\;+\;\frac{SD_{\text{2}}^{2}}{n_{2}}\right)}^{2}}{\frac{{\left(\frac{SD_{\text{1}}^{2}}{n_{1}}\right)}^{2}}{n_{1}-1}+\;\frac{{\left(\frac{SD_{\text{2}}^{2}}{n_{2}}\right)}^{2}}{n_{2}-1}}.$$

These equations are highly similar to the Student’s and Welch’s *t*-statistic for traditional significance tests. The only difference is that the lower equivalence bound Δ*_L_* and the upper equivalence bound Δ*_U_* are subtracted from the mean difference between groups. These bounds can be defined in raw scores or in a standardized difference, where Δ = Cohen’s *d* × σ or Cohen’s *d* = Δ/σ. The two one-sided tests are rejected if *t_U_* ≤ −*t*_(*df*, α)_, and *t_L_* ≥ *t*_(*df*, α)_, where *t*_(α, *df*)_ is the upper 100α percentile of a *t*-distribution (Berger & Hsu, 1996). The spreadsheet and R package can be used to perform this test, but some commercial software such as Minitab (Minitab 17 Statistical Software, 2010) also include the option to perform equivalence tests for *t* tests.

As an example, Eskine (2013) showed that participants who had been exposed to organic food were substantially harsher in their moral judgments relative to those in the control condition (*d* = .81, 95% CI [0.19, 1.45]). A replication by Moery and Calin-Jageman (2016, study 2) did not observe a significant effect (control: *n* = 95, *M* = 5.25, *SD* = .95, organic food: *n* = 89, *M* = 5.22, *SD* = .83). The authors followed Simonsohn’s (2015) recommendation so set the equivalence bound to the effect size the original study had 33% power to detect. With *n* = 21 in each condition of the original study, this means the equivalence bound is *d* = .48, which equals a difference of .384 on a 7-point scale given the sample sizes and a pooled *SD* of .894. We can calculate the TOST equivalence test *t*-values:

$$\frac{5.25-5.22-\left(-0.384\right)}{0.894\sqrt{\frac{1}{95}+\frac{1}{89}}}=t_{\text{L}}=3.14\text{ and }\frac{5.25-5.22-0.384}{0.894\sqrt{\frac{1}{95}+\frac{1}{89}}}=t_{\text{U}}=-2.69,$$

which correspond to *p* values of .001 and .004. If α = .05, and assuming equal variances, the equivalence test is significant, *t*(182) = −2.69, *p* = .004. We can reject effects larger than .384 scale points. Note that both one-sided tests need to be significant to declare equivalence; but for efficiency, only the one-sided test with the highest *p* value is reported in TOST results (given that if this test is significant, so is the other). Alternatively, because Moery and Calin-Jageman’s (2016) main prediction seems to be whether the effect is smaller than the upper equivalence bound (a test for inferiority), only the one-sided *t* test against the upper equivalence bound could be performed and reported. Note that the spreadsheet and R package allow you to either directly specify the equivalence bounds in Cohen’s *d* or set the equivalence bound in raw units.

An a priori power analysis for equivalence tests can be performed by calculating the required sample sizes to declare equivalence for two one-sided tests based on the lower equivalence bound and upper equivalence bound. When equivalence bounds are symmetric around zero (e.g., *Δ_L_* = −.5 and *Δ_U_* = .5), the required sample sizes (referred to as *n_L_* and *n_U_* in Equation 5) will be identical. Following Chow, Shao, and Wang (2002), the normal approximation of the power equation for equivalence tests (for each independent group of an independent *t* test) given a specific α level and desired level of statistical power (1 − β) is:

5 $$n_{\text{L}}=\frac{2{\left(z_{\alpha }+z_{\beta }/2\right)}^{2}}{\Delta_{\text{L}}{\;}^{2}},\;n_{\text{U}}=\frac{2{(z_{\alpha }+z_{\beta }/2)}^{2}}{\Delta_{\text{U}}{\;}^{2}},$$

where *Δ_L_* and *Δ_U_* are the standardized mean difference equivalence bounds (in Cohen’s *d*). This equation calculates the required sample sizes based on the assumption that the true effect size is zero (see Table 1). If a nonzero true effect size is expected, an iterative procedure must be used. A highly accessible overview of power analysis for equivalence, superiority, and noninferiority designs with power tables for a wide range of standardized mean differences and expected true mean differences that can be used to decide upon the sample size in your study is available in Julious’s (2004) study.

The narrower the equivalence bounds, or the smaller the effect sizes one tries to reject, the larger the sample size that is required. Large sample sizes are required to achieve high power when equivalence bounds are close to zero. This is comparable to the large sample sizes that are required to reject a true but small effect when the null hypothesis is a null effect. Equivalence tests require slightly larger sample sizes than traditional null hypothesis tests.

**Table 1.** Sample Sizes (for the Number of Observations in Each Group) for Equivalence Tests for Independent Means, as a Function of the Desired Power, α Level, and Equivalence Bound Δ (in Cohen’s *d*), Based on Exact Calculations and the Approximation.

Bound (Δ)	Approximation				Exact			
	80% Power		90% Power		80% Power		90% Power	
	α = .05	α = .01	α = .05	α = .01	α = .05	α = .01	α = .05	α = .01
0.1	1,713	2,604	2,165	3,155	1,713	2,604	2,165	3,155
0.2	429	651	542	789	429	652	542	789
0.3	191	290	241	351	191	291	242	351
0.4	108	163	136	198	108	165	136	199
0.5	69	105	87	127	70	106	88	128
0.6	48	73	61	88	49	74	61	89
0.7	35	54	45	65	36	55	45	66
0.8	27	41	34	50	28	43	35	51

## Equivalence Tests for Differences Between Dependent Means

When comparing dependent means, the correlation between the observations has to be taken into account, and the effect size directly related to the statistical significance of the test (and thus used in power analysis) is Cohen’s *d_z_* (see Lakens, 2013). The *t*-values for the two one-sided tests statistics are:

6 $$t_{\text{L}}=\frac{{\bar{M}}_{1}-{\bar{M}}_{2}-\Delta_{\text{L}}}{\frac{\sqrt{SD_{\text{1}}^{2}\;+\;SD_{\text{2}}^{2}-\;2\;\times \;r\;\times \;SD_{1}\;\times \;SD_{2}}}{\sqrt{N}}}\text{ and }t_{\text{U}}=\frac{{\bar{M}}_{1}-{\bar{M}}_{2}-\Delta_{\text{U}}}{\frac{\sqrt{SD_{\text{1}}^{2}\;+\;SD_{\text{2}}^{2}\;-\;2\;\times \;r\;\times \;SD_{1}\times \;SD_{2}}}{\sqrt{N}}}.$$

The bounds Δ*_L_* and Δ*_U_* can be defined in raw scores, or in a standardized bound based on Cohen’s *d_z_*, where Δ = *d_z_* × *SD*_diff_, or *d_z_* = Δ/*SD*_diff_. Equation 3 can be used for a priori power analyses by inserting Cohen’s *d_z_* instead of Cohen’s *d*. The number of pairs needed to achieve a desired level of power when using Cohen’s *d_z_* is half the number of observations needed in each between subject condition specified in Table 1.

There are no suggested benchmarks of small, medium, and large effects for Cohen’s *d_z_*. We can consider two approaches to determining benchmarks. The first is to use the same benchmarks for Cohen’s *d* as for Cohen’s *d_z_*. This assumes *r* = .5, when Cohen’s *d* and Cohen’s *d_z_* are identical.^3^ A second approach is to scale the benchmarks for Cohen’s *d_z_* based on the sample size we need to reliably detect an effect. For example, in an independent *t* test, 176 participants are required in each condition to achieve 80% power for *d* = .3 and α = .05. With 176 pairs of observations and α = .05, a study has 80% power for a Cohen’s *d_z_* of .212. The relationship between *d* and *d_z_* is a factor of $\sqrt{2}$, which means we can translate the benchmarks for Cohen’s *d* for small (.2), medium (.5), and large (.8) effects into benchmarks for Cohen’s *d_z_* of small (.14), medium (.35), and large (.57). There is no objectively correct way to set benchmarks for Cohen’s *d_z_*. I leave it up to the reader to determine whether either of these approaches is useful.

## Equivalence Tests for One-Sample *t* Tests

The *t*-values for the two one-sided tests for a one-sample *t* tests are:

7 $$t_{\text{L}}=\frac{M-\mu -\Delta_{\text{L}}}{\frac{S\text{D}}{\sqrt{N}}}\text{ and }t_{\text{U}}=\frac{M-\mu -\Delta_{\text{U}}}{\frac{S\text{D}}{\sqrt{N}}},$$

where *M* is the observed mean, *SD* is the observed standard deviation, *N* is the sample size, $\Delta_{\text{L}}$ and $\Delta_{\text{U}}$ are lower and upper equivalence bounds, and μ is the value that the mean is tested against.

## Equivalence Tests for Correlations

Equivalence tests can also be performed on correlations, where the two one-sided tests aim to reject correlations larger than a lower equivalence bound (*r_L_*) and smaller than an upper equivalence bound (*r_U_*). I follow Goertzen and Cribbie (2010), who use Fisher’s *z* transformation on the correlations, after which critical values are calculated that can be compared against the normal distribution:

8 $$Z_{\text{L}}=\;\frac{\frac{L\text{N}\left(\frac{1+r}{1-r}\right)}{2}-\frac{L\text{N}\left(\frac{1+r_{\text{L}}}{1-r_{\text{L}}}\right)}{2}}{\frac{1}{\sqrt{N-3}}},\;Z_{\text{U}}=\;\frac{\frac{L\text{N}\left(\frac{1+r}{1-r}\right)}{2}-\frac{L\text{N}\left(\frac{1+r_{\text{U}}}{1-r_{\text{U}}}\right)}{2}}{\frac{1}{\sqrt{N-3}}}.$$

The two one-sided tests are rejected if *Z_L_* ≤ −*Z*_α_ and *Z_U_* ≥ *Z*_α_. Benchmarks for small, medium, and large effects, which can be used to set equivalence bounds, are *r* = .1, *r* = .3, and *r* = .5. Power analysis for correlations can be performed by converting *r* to Cohen’s *d* using:

9 $$d=\;\frac{2r}{\sqrt{1-r^{2}}},$$

after which Equation 5 can be used. This approach is used by, for example, G*Power (Faul, Erdfelder, Lang, & Buchner, 2007).

## Equivalence Test for Meta-Analyses

Rejecting small effects in an equivalence test requires large samples. If researchers want to perform an equivalence test with narrow equivalence bounds (e.g., Δ*_L_* = −.1 and Δ*_U_* = .1), in most cases, only a meta-analysis will have sufficient statistical power. Rogers, Howard, and Vessey (1993) explain the straightforward approach to performing equivalence tests for meta-analyses:

10 $$Z_{\text{L}}=\;\frac{\text{Δ}+{\text{ Δ}}_{L}}{S\text{E}},\;Z_{\text{U}}=\;\frac{\text{Δ}+{\text{ Δ}}_{U}}{S\text{E}}.$$

where Δ is the meta-analytic effect size (Cohen’s *d* or Hedges’ *g*), and *SE* is the meta-analytic standard error (or $\sqrt{\text{var}}$). These values can be calculated with meta-analysis software such as metafor (Viechtbauer, 2010). The two one-sided tests are rejected if *Z_L_* ≤ −*Z*_α_ and *Z_U_* ≤ *Z*_α_. Alternatively, the 90% CI can be reported. If the 90% CI falls within the equivalence bounds, the observed meta-analytic effect is statistically equivalent.

## Setting Equivalence Bounds

In psychology, most theories do not state which effects are too small to be interpreted as support for the proposed underlying mechanism. Instead, feasibility considerations are often the strongest determinant of the effect sizes a researcher can reliably examine. In daily practice, researchers have a maximum sample size they are willing to collect in a single study (e.g., 100 participants in each between-subject condition). Given a desired level of statistical power (e.g., 80%) and a specific α (e.g., .05), this implies a smallest effect size they find worthwhile to examine or a SESOI (Lakens, 2014) they can reliably examine. Based on a sensitivity analysis in power analysis software (such as G*Power), we can calculate that with 100 participants in each condition, 80% desired power, and an α of .05, the SESOI in a null effect significance test is Δ = 0.389; and using the power analysis calculation for an equivalence test for independent samples, assuming a true effect size of 0, 80% power is achieved when Δ*_L_* = −0.414 and Δ*_U_* = 0.414. As such, without practical boundaries or theoretical boundaries that indicate which effect size is meaningful, the maximum sample size you are willing to collect implicitly determines your SESOI. Therefore, setting equivalence boundaries to your SESOI in an equivalence test allows you to reject effect sizes larger than you find worthwhile to examine, given available resources. When researchers are not willing (or not able) to collect a decent sample size, the extremely large equivalence bounds will make it clear they can at best reject extremely large effects, but that their data are not informative about the presence or absence of a wide range of plausible and interesting effect sizes.

This recommendation differs from practices in drug development, where equivalence bounds are often set by regulations (e.g., differences up to 20% are not considered to be clinically relevant). In psychology, such general regulations about what constitutes a meaningful effect seem unlikely to emerge and perhaps even undesirable. Using equivalence bounds based on effect sizes a researcher finds worthwhile to examine do not allow psychologists to conclude an effect is too small to be meaningless *for anyone*. When other researchers believe a smaller effect size is plausible and theoretically interesting, they can design a study with a larger sample size to examine the effect. In randomized controlled trials, it is expected that equivalence bounds are prespecified (e.g., see CONSORT guidelines; Piaggio et al., 2006), and this should also be considered best practice in psychology. When in the abstract of an article, authors conclude an effect is “statistically equivalent,” the abstract should also include the equivalence bounds that are used to draw this conclusion.

Simonsohn (2015) proposes to test for inferiority for replication studies (an equivalence test where the lower bound is set to infinity). He suggests to set the upper equivalence bound in a replication study to the effect size that would have given an original study 33% power. For example, an original study with 60 participants divided equally across two independent groups has 33% power to detect an effect of *d* = .4, so Δ*_U_* is set to *d* = .4. This approach limits the sample size required to test for equivalence to 2.5 times the sample size of the original study. The goal is not to show the effect is too small to be feasible to study but too small to have been reliably detected by the original experiment, thus casting doubt on the original observation.

If feasibility constraints are practically absent (e.g., in online studies), another starting point to set equivalence bounds is by setting bounds based on benchmarks for small, medium, and large effects. Although using these benchmarks to interpret effect sizes is typically recommended as a last resort (e.g., Lakens, 2013), their use in setting equivalence bounds seems warranted by the lack of other clear-cut recommendations. By far the best solution would be for researchers to specify their SESOI when they publish an original result or describe a theoretical idea (Morey & Lakens, 2017). The use of equivalence testing will no doubt lead to a discussion about which effect sizes are too small to be worthwhile to examine in specific research lines in psychology, which in itself is progress.

## Discussion

Equivalence tests are a simple adaptation of traditional significance tests that allow researchers to design studies that reject effects larger than prespecified equivalence bounds. It allows researchers to reject effects large enough to be considered worthwhile. Adopting equivalence tests will prevent the common misinterpretations of nonsignificant *p* values as the absence of an effect and nudge researchers toward specifying which effects they find worthwhile. By providing a simple spreadsheet and R package to perform power calculations and equivalence tests for common statistical tests in psychology, researchers should be able to easily improve their research practices.

Rejecting effects more extreme than the equivalence bounds implies that we can conclude equivalence for a specific operationalization of a hypothesis. It is possible that a meaningful effect would be observed with a different manipulation or measure. Confounds can underlie observed equivalent effects. An additional nonstatistical challenge in interpreting equivalence concerns the issue of whether an experiment was performed competently (Senn, 2007). Complete transparency (sharing all materials) is a partial solution since it allows peers to evaluate whether the experiment was well designed (Morey et al., 2016), but this issue is not easily resolved when the actions of an experimenter might influence the data. In such experiments, even blinding the experimenter to conditions is no solution since an experimenter can interfere with the data quality of all conditions. This is an inherent asymmetry between demonstrating an effect and demonstrating the absence of a worthwhile effect. The only solution for anyone skeptical about studies demonstrating equivalence is to perform an independent replication.

Equivalence testing is based on a Neyman–Pearson hypothesis testing approach that allows researchers to control error rates in the long run and design studies based on a desired level of statistical power. Error rates in equivalence tests are controlled at the α level when the true effect equals the equivalence bound. When the true effect is more extreme than the equivalence bounds, error rates are smaller than the α level. It is important to take statistical power into account when determining the equivalence bounds because, in small samples (where CIs are wide), a study might have no statistical power (i.e., the CI will always be so wide that it is necessarily wider than the equivalence bounds).

There are alternative approaches to the TOST procedure. Updated versions of equivalence tests exist, but their added complexity does not seem to be justified by the small gain in power (for a discussion, see Meyners, 2012). There are also alternative approaches to providing statistical support for a small or null effect, such as estimation (calculating effect sizes and CIs), specifying a region of practical equivalence (Kruschke, 2010), or calculating Bayes factors (Dienes, 2014; Rouder, Speckman, Sun, Morey, & Iverson, 2009). Researchers should report effect size estimates in addition to hypothesis tests. Since Bayesian and frequentist tests answer complementary questions, with Bayesian statistics quantifying posterior beliefs, and Frequentist statistics controlling Type 1 and Type 2 error rates, these tests can be reported side by side.

Other fields are able to use raw measures due to the widespread use of identical measurements (e.g., the number of deaths, the amount of money spent), but in some subfields in psychology the variability in the measures that are collected require standardized effect sizes to make comparisons across studies (Cumming & Fidler, 2009). A consideration of using standardized effect sizes as equivalence bounds is that in two studies with the same mean difference and CIs in raw scale units (e.g., a difference of 0.2 on a 7-point scale with 90% CI [−0.13;0.17]), the same standardized equivalence bounds can lead to different significance levels in a equivalence test. The reason for this is that the pooled *SD* can differ across the studies, and as a consequence, the same equivalence bounds in standardized scores imply different equivalence bounds in raw scores. If this is undesirable, researchers should specify equivalence bounds in raw scores instead.

Ideally, psychologists could specify equivalence bounds in raw mean differences based on theoretical predictions or cost–benefit analyses, instead of setting equivalence bounds based on standardized benchmarks. My hope is that as equivalence tests become more common in psychology, researchers will start to discuss which effect sizes are theoretically expected while setting equivalence bounds. When theories do not specify which effect sizes are too small to be meaningful, theories can’t be falsified. Whenever a study yields no statistically significant effect, one can always argue that there is a true effect that is smaller than the study could reliably detect (Morey & Lakens, 2017). Maxwell, Lau, and Howard (2015) suggest that replication studies demonstrate the absence of an effect by using equivalence bounds of Δ*_L_* = −.1 and Δ*_U_* = .1 or even Δ*_L_* = −.05 and Δ*_U_* = .05. I believe this creates an imbalance where we condone original studies that fail to make specific predictions, while replication studies are expected to test extremely specific predictions that can only be confirmed by collecting huge numbers of observations.

Extending your statistical tool kit with equivalence tests is an easy way to improve your statistical and theoretical inferences. The TOST procedure provides a straightforward approach to reject effect sizes that one considers large enough to be worthwhile to examine.

## Notes

1. As Wellek (2010, p. 30) notes, for all practical purposes, one can simply specify a very large value for the infinite equivalence bound.

2. A 90% confidence interval (CI; 1 − 2α) is used instead of a 95% CI (1 − α) because two one-sided tests (each with an α of 5%) are performed.

3. The author would like to thank Jake Westfall for this suggestion.

## References

[bibr1-1948550617697177] Anderson S. F. Maxwell S. E. (2016). There’s more than one way to conduct a replication study: Beyond statistical significance. Psychological Methods, 21, 1–12. doi:https://doi.org/11037/met0000051 26214497 10.1037/met0000051

[bibr2-1948550617697177] Berger R. L. Hsu J. C. (1996). Bioequivalence trials, intersection-union tests and equivalence confidence sets. Statistical Science, 11, 283–302.

[bibr3-1948550617697177] Chow S.-C. Shao J. Wang H. (2002). A note on sample size calculation for mean comparisons based on noncentral *t*-statistics. Journal of Biopharmaceutical Statistics, 12, 441–456. 12477068 10.1081/BIP-120016229

[bibr4-1948550617697177] Cumming G. Fidler F. (2009). Confidence intervals: Better answers to better questions. Zeitschrift Für Psychologie/Journal of Psychology, 217, 15–26. doi:10.1027/0044-3409.217.1.15

[bibr5-1948550617697177] Cumming G. Finch S. (2001). A primer on the understanding, use, and calculation of confidence intervals that are based on central and noncentral distributions. Educational and Psychological Measurement, 61, 532–574. doi:10.1177/0013164401614002

[bibr6-1948550617697177] Delacre M. Lakens D. Leys C. (2017). Why psychologists should by default use Welch’s t-test instead of Student’s t-test with unequal group sizes. International Review of Social Psychology, 30, 92–101. doi:10.5334/irsp.82

[bibr7-1948550617697177] Dienes Z. (2014). Using Bayes to get the most out of non-significant results. Quantitative Psychology and Measurement, 5, 781 doi:10.3389/fpsyg.2014.00781 10.3389/fpsyg.2014.00781 PMC4114196 25120503

[bibr8-1948550617697177] Dienes Z. (2016). How Bayes factors change scientific practice. Journal of Mathematical Psychology. doi:10.1016/j.jmp.2015.10.003 10.3389/fpsyg.2016.01504 PMC5043379 27775731

[bibr9-1948550617697177] Eskine K. J. (2013). Wholesome foods and wholesome morals? Organic foods reduce prosocial behavior and harshen moral judgments. Social Psychological and Personality Science, 4, 251–254. doi:10.1177/1948550612447114

[bibr10-1948550617697177] Faul F. Erdfelder E. Lang A.-G. Buchner A. (2007). G* Power 3: A flexible statistical power analysis program for the social, behavioral, and biomedical sciences. Behavior Research Methods, 39, 175–191. 17695343 10.3758/bf03193146

[bibr11-1948550617697177] Finch S. Cumming G. Thomason N. (2001). Colloquium on effect sizes: The roles of editors, textbook authors, and the publication manual reporting of statistical inference in the journal of applied psychology: Little evidence of reform. Educational and Psychological Measurement, 61, 181–210. doi:10.1177/0013164401612001

[bibr12-1948550617697177] Gigerenzer G. (1998). Surrogates for theories. Theory and Psychology, 8, 195–204.

[bibr13-1948550617697177] Goertzen J. R. Cribbie R. A. (2010). Detecting a lack of association: An equivalence testing approach. British Journal of Mathematical and Statistical Psychology, 63, 527–537. doi:10.1348/000711009X475853 20030968 10.1348/000711009X475853

[bibr14-1948550617697177] Hauck D. W. W. Anderson S. (1984). A new statistical procedure for testing equivalence in two-group comparative bioavailability trials. Journal of Pharmacokinetics and Biopharmaceutics, 12, 83–91. doi:10.1007/BF01063612 6747820 10.1007/BF01063612

[bibr15-1948550617697177] Julious S. A. (2004). Sample sizes for clinical trials with normal data. Statistics in Medicine, 23, 1921–1986. doi:10.1002/sim.1783 15195324 10.1002/sim.1783

[bibr16-1948550617697177] Kruschke J. (2010). Doing Bayesian data analysis: A tutorial introduction with R. Burlington, MA: Academic Press.

[bibr17-1948550617697177] Lakens D. (2013). Calculating and reporting effect sizes to facilitate cumulative science: A practical primer for t-tests and ANOVAs. Frontiers in Psychology, 4 doi:10.3389/fpsyg.2013.00863 10.3389/fpsyg.2013.00863 PMC3840331 24324449

[bibr18-1948550617697177] Lakens D. (2014). Performing high-powered studies efficiently with sequential analyses: Sequential analyses. European Journal of Social Psychology, 44, 701–710. doi:10.1002/ejsp.2023

[bibr19-1948550617697177] Maxwell S. E. Kelley K. Rausch J. R. (2008). Sample size planning for statistical power and accuracy in parameter estimation. Annual Review of Psychology, 59, 537–563. doi:10.1146/annurev.psych.59.103006.093735 10.1146/annurev.psych.59.103006.093735 17937603

[bibr20-1948550617697177] Maxwell S. E. Lau M. Y. Howard G. S. (2015). Is psychology suffering from a replication crisis? What does “failure to replicate” really mean? American Psychologist, 70, 487–498. doi:10.1037/a0039400 26348332 10.1037/a0039400

[bibr21-1948550617697177] Meyners M. (2012). Equivalence tests—A review. Food Quality and Preference, 26, 231–245. doi:10.1016/j.foodqual.2012.05.003

[bibr38-1948550617697177] Minitab 17 Statistical Software. (2010). [Computer software]. State College, PA: Minitab, Inc.

[bibr22-1948550617697177] Moery E. Calin-Jageman R. J. (2016). Direct and conceptual replications of Eskine (2013): Organic food exposure has little to no effect on moral judgments and prosocial behavior. Social Psychological and Personality Science, 7, 312–319. doi:10.1177/1948550616639649

[bibr23-1948550617697177] Morey R. D. Chambers C. D. Etchells P. J. Harris C. R. Hoekstra R. Lakens D.…Zwaan R. A. (2016). The peer reviewers’ openness initiative: Incentivizing open research practices through peer review. Royal Society Open Science, 3, 150547. 26909182 10.1098/rsos.150547 PMC4736937

[bibr24-1948550617697177] Morey R. D. Lakens D. (2017). Why most of psychology is statistically unfalsifiable. Manuscript submitted for publication.

[bibr25-1948550617697177] Piaggio G. Elbourne D. R. Altman D. G. Pocock S. J. Evans S. J. Group C. (2006). Reporting of noninferiority and equivalence randomized trials: An extension of the CONSORT statement. Journal of the American Medical Association, 295, 1152–1160. 16522836 10.1001/jama.295.10.1152

[bibr26-1948550617697177] Quertemont E. (2011). How to statistically show the absence of an effect. Psychologica Belgica, 51, 109 doi:10.5334/pb-51-2-109

[bibr27-1948550617697177] Rogers J. L. Howard K. I. Vessey J. T. (1993). Using significance tests to evaluate equivalence between two experimental groups. Psychological Bulletin, 113, 553. 8316613 10.1037/0033-2909.113.3.553

[bibr28-1948550617697177] Rouder J. N. Speckman P. L. Sun D. Morey R. D. Iverson G. (2009). Bayesian t tests for accepting and rejecting the null hypothesis. Psychonomic Bulletin & Review, 16, 225–237. doi:10.3758/PBR.16.2.225 19293088 10.3758/PBR.16.2.225

[bibr29-1948550617697177] Ruxton G. D. (2006). The unequal variance t-test is an underused alternative to Student’s t-test and the Mann-Whitney U test. Behavioral Ecology, 17, 688–690. doi:10.1093/beheco/ark016

[bibr39-1948550617697177] Satterthwaite F. E. (1946). An approximate distribution of estimates of variance components. Biometrics Bulletin, 2, 110–114. doi:10.2307/3002019 20287815

[bibr30-1948550617697177] Schuirmann D. J. (1987). A comparison of the two one-sided tests procedure and the power approach for assessing the equivalence of average bioavailability. Journal of Pharmacokinetics and Biopharmaceutics, 15, 657–680. 3450848 10.1007/BF01068419

[bibr31-1948550617697177] Seaman M. A. Serlin R. C. (1998). Equivalence confidence intervals for two-group comparisons of means. Psychological Methods, 3, 403–411. doi:10.1037/1082-989X.3.4.403

[bibr32-1948550617697177] Senn S. (2007). Statistical issues in drug development (2nd ed.). Hoboken, NJ: Wiley.

[bibr34-1948550617697177] Simonsohn U. (2015). Small telescopes detectability and the evaluation of replication results. Psychological Science, 26, 559–569. doi:10.1177/0956797614567341 25800521 10.1177/0956797614567341

[bibr35-1948550617697177] Viechtbauer W. (2010). Conducting meta-analyses in R with the metafor package. Journal of Statistical Software, 36, 1–48.

[bibr36-1948550617697177] Weber R. Popova L. (2012). Testing equivalence in communication research: Theory and application. Communication Methods and Measures, 6, 190–213. doi:10.1080/19312458.2012.703834

[bibr40-1948550617697177] Welch B. L. (1947). The generalization of student’s’ problem when several different population variances are involved. Biometrika, 34, 28–35. doi:10.2307/2332510 20287819 10.1093/biomet/34.1-2.28

[bibr37-1948550617697177] Wellek S. (2010). Testing statistical hypotheses of equivalence and noninferiority (2nd ed.). Boca Raton, FL: CRC Press.

